# Simple hepatic cyst is not always simple – a case report of cystobiliary communication requiring hepatic resection

**DOI:** 10.1097/MS9.0000000000005656

**Published:** 2026-09-14

**Authors:** Chin-Yee Mah, Chang-Kuo Wei, Chun-Hung Lin, Shih-Pin Lin, Yu-Ting Chiu, Wen-Yao Yin, Cheng-Hung Lee

**Affiliations:** a Department of General Surgery, Buddhist Dalin Tzu Chi General Hospital, Chia-Yi, Taiwan; b Department of Surgery, School of Medicine, Tzu Chi University, Hualian, Taiwan

**Keywords:** bile content, hepatectomy, hepatic resection, huge liver cyst, simple hepatic cyst

## Abstract

**Background:**

Simple hepatic cysts are common and usually asymptomatic, but rarely may communicate with the intrahepatic biliary tree; unroofing alone can then result in postoperative bile leakage if this communication is not recognized.

**Case presentation:**

We report a 74-year-old woman with multiple hepatic cysts, the largest measuring 11 cm and causing extrinsic gastric compression. Preoperative computed tomography (CT) suggested a simple cyst without evidence of biliary communication, and laparoscopic unroofing was planned. Needle decompression, however, yielded bile-like fluid indicating unsuspected cystobiliary communication; the procedure was therefore converted to laparoscopic left lateral sectionectomy. Her postoperative course was uneventful, without bile leakage.

**Conclusion:**

In conclusion, a preoperatively “simple” hepatic cyst may harbor unsuspected cystobiliary communication that CT alone can miss. Intraoperative vigilance and willingness to convert to formal resection are essential when this complication is encountered.

## Introduction

Simple hepatic cysts are common incidental findings on abdominal imaging, with a calculated incidence of 4.75% on ultrasonography, and they mostly remain asymptomatic throughout a patient’s lifetime[1]. Complications include mass effect, rupture, hemorrhage, or infection[2]. Cystobiliary communication is a rare complication that requires careful selection of treatment to prevent further unfavorable outcomes[3]. This case highlights the importance of recognizing when a “simple” hepatic cyst may harbor unexpected complications requiring more aggressive surgical management. We aim to describe this presentation, highlight diagnostic pitfalls that may cause cystobiliary communication to be missed on preoperative cross-sectional imaging, and discuss the intraoperative decision-making and surgical alternatives once biliary communication is identified.

HIGHLIGHTS

- Simple hepatic cysts are common, but rare cystobiliary communication may not be evident on preoperative computed tomography.
- Bile-like fluid on needle decompression should raise immediate suspicion for cystobiliary communication.
- Simple cyst unroofing risks postoperative bile leakage when an unrecognized biliary communication is present.
- Laparoscopic anatomical resection is a safe and effective alternative when a communication is confirmed intraoperatively.
- Intraoperative vigilance and willingness to modify the surgical plan are key to optimal outcomes.

## Case report

A 74-year-old female presented with a 2-year history of epigastric discomfort that had recently progressed to postprandial fullness. Physical examination revealed a palpable left upper quadrant mass. On admission, she was afebrile and hemodynamically stable (temperature 36°C, blood pressure 115/65 mmHg, heart rate 78 beats/min, respiratory rate 16 breaths/min). Abdominal sonography showed multiple simple hepatic cysts in both lobes. Esophagogastroduodenoscopy (EGD) showed that the lesser curvature side of the gastric body was compressed by an external mass lesion (Fig. 1). Laboratory findings disclosed mildly elevated aspartate transaminase (42 IU/L), alanine transaminase (68 IU/L), alkaline phosphatase (265 IU/L), and hyperbilirubinemia (total bilirubin 1.08 mg/dL, direct bilirubin 0.31 mg/dL). Renal function and coagulation parameters were normal, and there was no leukocytosis. A computed tomography (CT) scan demonstrated multiple bilobar liver cysts. The largest was an 11 cm, well-defined hepatic cyst without internal contents that occupied the lateral segment, causing external compression of the stomach (Fig. 2). There was neither infiltration nor ascites around the left lobe cyst.

**Figure 1.**
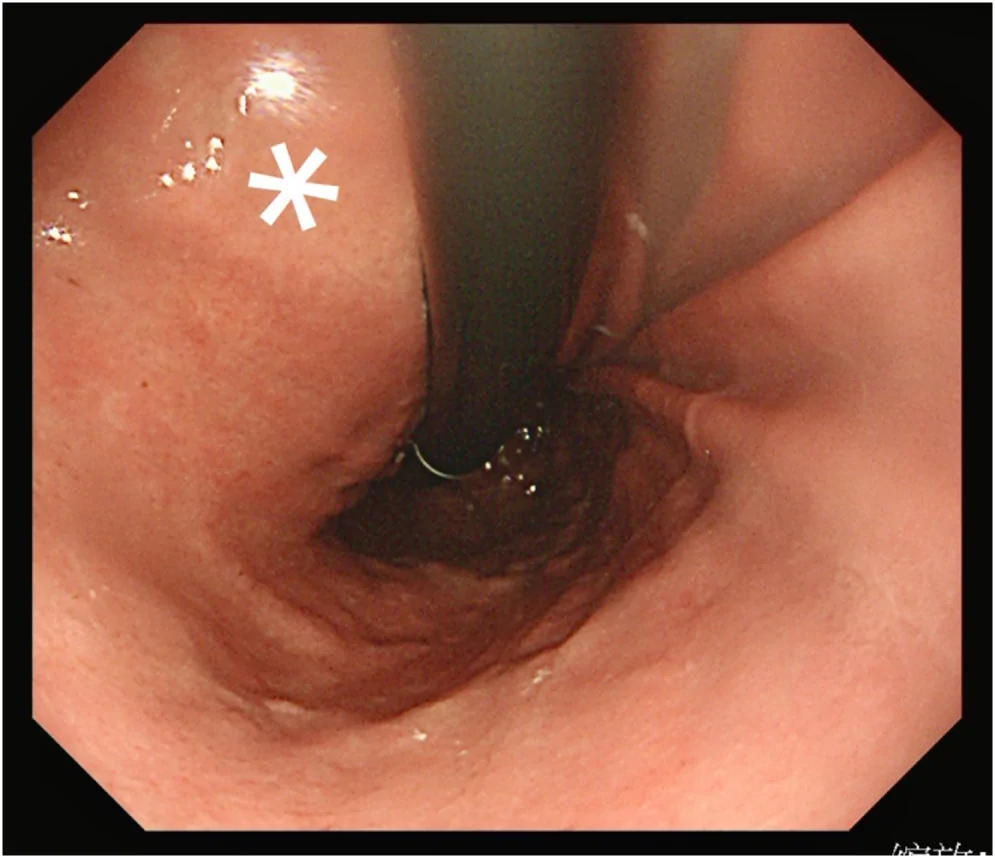
EGD revealed that the lesser curvature of the stomach (area marked with an asterisk) was externally compressed, explaining her postprandial fullness.

**Figure 2.**
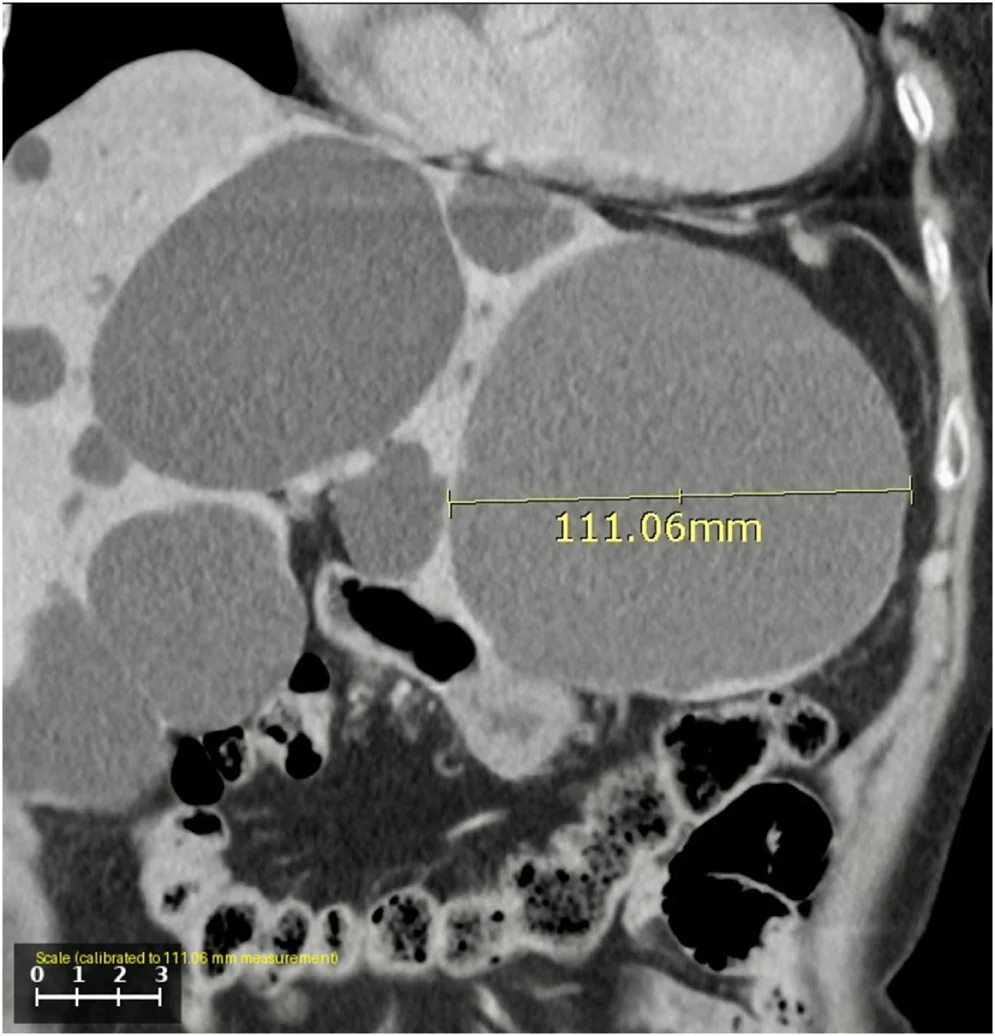
Contrast-enhanced coronal CT showed multiple hepatic cysts, with the largest located in the left lobe. The stomach was displaced inferiorly by the large left-lobe cyst. The largest cyst measured 11 cm at its greatest dimension on CT.

Timeline: The patient’s epigastric discomfort had been present for approximately 2 yr and progressed to postprandial fullness in the 3 months preceding admission, prompting evaluation. Physical examination, laboratory testing, EGD, and CT were performed before admission (Figs 1–2). Laparoscopic surgery was performed on hospital day 1; intraoperative needle decompression revealed bile-like fluid, and the procedure was converted to a laparoscopic left lateral sectionectomy. The postoperative course was uneventful, and the patient was discharged on postoperative day 6.

Preoperative imaging suggested that the lesion was a simple hepatic cyst responsible for the patient’s symptoms. Laparoscopic unroofing of the hepatic cyst was planned. Needle cyst decompression was performed as the initial step. Upon puncture, a viscous, high-pressure, bile-containing fluid was expelled rapidly from the small puncture site (Fig. 3). This finding was highly suggestive of cystobiliary communication. Post-operative bile leakage could occur if simple unroofing were performed. A laparoscopic left lateral sectionectomy was chosen to ensure complete cyst excision and bile duct closure to prevent bile leakage. During surgery, multiple small hepatic cysts were encountered along the resection line. They were opened and unroofed if needed. All were simple cysts without bile. Pathology reported a benign biliary cyst. The patient had an uneventful postoperative recovery without bile leakage and experienced significant symptomatic relief.

**Figure 3.**
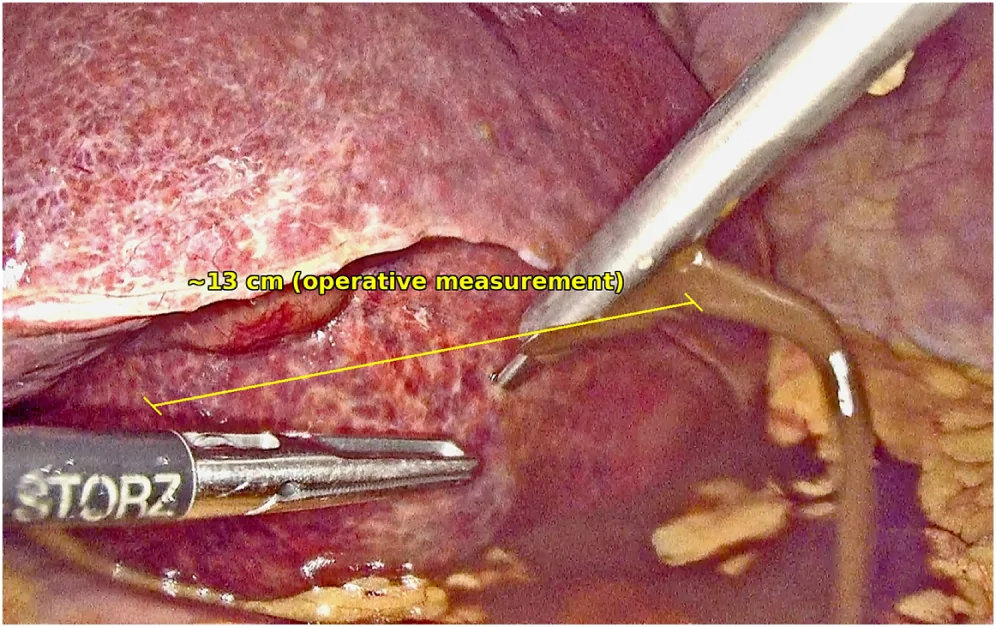
Viscid biliary fluid spurted out from a small needle puncture site. The operative field was contaminated by cyst contents. The appearance of viscid, bile-like fluid upon needle puncture was diagnostic of cystobiliary communication and prompted intraoperative conversion from the planned cyst unroofing to laparoscopic left lateral sectionectomy to allow definitive closure of the communicating bile duct.

## Discussion

Most hepatic cysts are simple cysts, which arise from ductal plate malformations during embryonic development. When portions of the ductal plate disconnect from the biliary tree, they can develop into cystic structures. The epithelial cells lining these cysts retain secretory function, producing fluid that mirrors bile composition but typically lacks bile acids and bilirubin – unless cystobiliary communication develops[4]. Cystobiliary communication is exceedingly rare in simple hepatic cysts, whereas it is well documented in hydatid disease, where it occurs in 14–37% of cases[3].

According to the European Association for the Study of the Liver (EASL) Clinical Practice Guidelines in 2022, ultrasound is used for the initial diagnosis of simple hepatic cysts. Hepatic cysts with complex features, including an atypical cyst wall or contents, require CT or MRI to evaluate cyst distribution, assess the relationship to the hepatic vasculature, and evaluate for hemorrhage or infection[4]. Moreover, in pre-operative diagnosis, magnetic resonance cholangiopancreatography (MRCP) provides a comprehensive evaluation of the biliary anatomy, identifies communication between cysts and the bile ducts, and detects bile duct dilatation caused by cyst compression or obstruction. This detailed anatomical mapping is essential for surgical planning and helps minimize the risk of bile duct injury. Endoscopic retrograde cholangiopancreatography (ERCP) offers both diagnostic and therapeutic capabilities. Diagnostically, ERCP can directly visualize and confirm cystobiliary communication, identify the precise location of bile leakage, and obtain cytology or tissue samples for pathology. Therapeutically, ERCP enables drainage of cystobiliary communications through the placement of drainage catheters or stents, and endoscopic sphincterotomy, to improve bile flow[3]. Another simple but invasive diagnostic method is percutaneous pigtail drainage, with cyst fluid sent for analysis. In our case, we did not perform MRI or invasive ERCP because a simple hepatic cyst, rather than a complicated cyst, was favored by preoperative imaging. It was not until we punctured the cyst that cystobiliary communication was diagnosed.

CT can under-detect cystobiliary communication for several reasons. Communicating channels are frequently submillimeter in caliber and effectively invisible at conventional CT resolution, and communication may be intermittent, becoming apparent only when intracystic pressure exceeds biliary pressure. Bile-containing cyst fluid can also be isodense with simple serous fluid, and cyst walls may be difficult to separate from adjacent parenchyma, which limits the specificity of CT for characterizing hepatic cystic lesions[5]. Features that should raise suspicion for biliary communication include a thickened or irregular cyst wall, internal septations, mild upstream ductal dilation, and apparent contiguity between the cyst and a bile duct on multiplanar reformats. In our patient, mildly elevated liver enzymes and bilirubin on admission were nonspecific and were not recognized preoperatively as a possible clue to biliary involvement; in retrospect, such biochemical derangement in the context of a large hepatic cyst may warrant a lower threshold for MRCP.

For biliary cysts, several case reports describe different management strategies for hepatic cysts with biliary communication. Laparoscopic deroofing has been reported as a viable option even for giant hepatic cysts, provided that the biliary communication is recognized intraoperatively and the leakage site is managed through laparoscopic suturing[6]. Diagnostic delay is common in this condition because clinical presentations are nonspecific, and communication is often identified only intraoperatively rather than on preoperative imaging, as occurred in our patient[7]. Where enteric biliary decompression has been attempted, cystojejunostomy has been reported to carry a risk of ascending cholangitis from enteric contamination of a previously sterile cyst, occasionally requiring further surgery; wide resection of the cyst wall with primary closure of the communicating duct has therefore been proposed by some authors as a more definitive alternative to enteric drainage[7]. Endoscopic ultrasound-guided drainage is an emerging minimally invasive alternative, particularly useful in carefully selected patients who may not be ideal candidates for surgery[8]. In contrast, hepatectomy remains the definitive treatment for more complicated cases, especially polycystic liver disease with cystobiliary communication, where resection is often required to prevent recurrence or ongoing biliary complications[3]. Although hepatectomy can provide a definitive treatment, it may cause hepatic decompensation if a massive resection is performed. Thus, surgical decisions may vary from patient to patient. In our case, once cystobiliary communication was found during surgery, we recognized that simple unroofing would be inadequate, opting for definitive resection to prevent postoperative bile leakage. Owing to the cyst’s location, laparoscopic unroofing surgery can be changed to a laparoscopic lateral sectionectomy without conversion.

In conclusion, our case demonstrates that simple hepatic cysts are not always straightforward, particularly when they are complicated by cystobiliary communication. This case reinforces the importance of thorough preoperative evaluation, intraoperative vigilance, and a willingness to modify surgical plans based on unexpected findings to achieve optimal patient outcomes.

## Data Availability

The de-identified clinical data, imaging, and photographs supporting the findings of this case report are available from the corresponding author upon reasonable request.

## Research registration unique identifying number (UIN)

Not applicable – registration is not required for a single case report.
